# Exergy Analysis of the Musculoskeletal System Efficiency during Aerobic and Anaerobic Activities

**DOI:** 10.3390/e20020119

**Published:** 2018-02-11

**Authors:** Gabriel Marques Spanghero, Cyro Albuquerque, Tiago Lazzaretti Fernandes, Arnaldo José Hernandez, Carlos Eduardo Keutenedjian Mady

**Affiliations:** 1 School of Mechanical Engineering, University of Campinas, 13083-970 Campinas, Brazil; 2 Department of Mechanical Engineering, Centro Universitário da FEI, 09850-901 São Bernardo do Campo, Brazil; 3 Sports Medicine Group of the Department of Orthopedics and Traumatology, University of São Paulo Medical School, 01246-000 São Paulo, Brazil

**Keywords:** exergy analysis, human body, physical activities, metabolic efficiency, exergy efficiency

## Abstract

The first and second laws of thermodynamics were applied to the human body in order to evaluate the quality of the energy conversion during muscle activity. Such an implementation represents an important issue in the exergy analysis of the body, because there is a difficulty in the literature in evaluating the performed power in some activities. Hence, to have the performed work as an input in the exergy model, two types of exercises were evaluated: weight lifting and aerobic exercise on a stationary bicycle. To this aim, we performed a study of the aerobic and anaerobic reactions in the muscle cells, aiming at predicting the metabolic efficiency and muscle efficiency during exercises. Physiological data such as oxygen consumption, carbon dioxide production, skin and internal temperatures and performed power were measured. Results indicated that the exergy efficiency was around 4% in the weight lifting, whereas it could reach values as high as 30% for aerobic exercises. It has been shown that the stationary bicycle is a more adequate test for first correlations between exergy and performance indices.

## 1. Introduction

Exergy analysis is applied to the human body and to metabolism in order to evaluate the quality of the energy conversion process in the human body and in human muscle cells during some physical activities. The purpose of these analyses is to better understand muscle efficiency from the perspective of the second law of thermodynamics.

The second law of thermodynamics demonstrates that wherever there is an energy transfer with a disequilibrium, there must be some impact on the environment. Nevertheless, as proposed by [1], it is this imbalance that guarantees life. Moreover, all biological systems have their entropy at maximum when they approach death. A few decades later, [2] demonstrated that all living things tend to a minimum entropy production level. This makes sense, since the entropy of the body is closer to the environment (lower disequilibrium).

Different forms of applying exergy analysis have been studied in several control volumes over the past decades. Some authors evaluated a cancerous cell [3] or how these energy conversion processes occur at the metabolic scale [4,5]. These analyses were extended in order to evaluate the entropy generation in the human body in several conditions [6,7,8,9,10,11,12,13]. Most of these authors aimed to demonstrate the Prigogine principle.

Over time, new applications emerged, in order to evaluate thermal comfort conditions [14,15,16,17,18,19,20,21,22,23]. These analyses related the destroyed exergy and exergy transfer to the environment with the predicted mean vote. Later, some applications in medicine were found in the literature for the whole body [24,25], in organs or systems [25,26,27,28] and in sports or muscle evaluation [29,30,31,32,33]. There is even a review that discusses the importance of exergy analysis in each research field [34].

The aim of this analysis is to propose quality indicators based on exergy analysis (destroyed exergy and exergy efficiency), concerned with comparing it to the traditional indexes used in sports (maximum oxygen consumption and lactate threshold), which was already performed in Mady et al. [29]. An issue found in this work, with the subjects running on a treadmill, was in the evaluation of the thermodynamic definition of performed work (or power). Moreover, the different equations to obtain the performed power in the literature lead to values from 343 to 1650 W for speeds between 3.6 and 3.9 m/s, as discussed by [35].

It is important to highlight that the application of energy and exergy analysis to the human body in different conditions was already validated and extensively used in the literature by different authors [5,11,12,13,14,15,16,17,18,19,20,21,22,23,25,26,29,30,31,32,33,36]. The distinguishing feature of this article is the application to actual experimental results as in [5] for sports purposes and with the actual performed power known as an input variable. It is noteworthy that Mady et al. [36] evaluated different equations for performed work in order to compare the internal temperature obtained from the first law of thermodynamics with the measured tympanic temperature. Their results were inconclusive, but the simplest equation (body as a material point) led to better results. Hence, one of the major objectives of this analysis is to choose one type of exercise to better evaluate the energy and exergy analysis of athletes and then extend, in the future, to other activities. For the sake of simplicity, this article demonstrates an initial method where only two types of exercise (with two subjects) were investigated to better select in future experiments the most suitable experiment concerning the application of exergy analysis terms as a possible index of performance in sports. Regarding metabolism, one objective is the evaluation of the amount of aerobic and anaerobic oxidation of energy substrates. However, it is just an initial discussion of the contribution of these quantities to global metabolism. The purpose is to evaluate the amount of anaerobic oxidation without the use of the concept of maximum oxygen consumption and lactate threshold; this last one must be obtained by blood sample during the activity [5].

## 2. Thermodynamic Model

The first law of thermodynamics used in the present work is derived from Equation (1) and the exergy analysis from Equation (2) (for a given environmental/reference temperature $T_{0}$, pressure $p_{0}$ and relative humidity $\phi_{0}$).

(1) $$\frac{dU}{dt}=\sum \left({\dot{m}}_{in}h_{in}\right)-\sum {\dot{m}}_{out}h_{out}+{\dot{Q}}_{VC}-{\dot{W}}_{VC}$$

Equation (2) indicates the exergy analysis of a control volume with several inputs, outputs and heat transfer rates. It is important to point out that **B** is the exergy of the control volume obtained in Joules (J), whereas $\dot{B}=\dot{m_{i}}b_{i}$ is the exergy associated with the mass flow rate in Watts (W).

(2) $$\frac{dB}{dt}=\sum \left({\dot{m}}_{in}b_{in}\right)-\sum \left({\dot{m}}_{out}b_{out}\right)+\sum {\dot{Q}}_{VC}\left(1-\frac{T_{0}}{T_{VC}}\right)-{\dot{W}}_{VC}-{\dot{B}}_{d}$$

In these equations, $\frac{dU}{dt}$ stands for the internal energy variation of the body over time (W) and $\frac{dB}{dt}$ stands for the exergy variation of the body over time (W). The term $\dot{m}$ is the mass flow rate (kg/s); *h* is the specific enthalpy (kJ/kg); *b* the specific exergy (kJ/kg); ${\dot{Q}}_{VC}$ is the heat transfer rate (W) at a surface at $T_{VC}$; ${\dot{W}}_{VC}$ is the performed power (W); and ${\dot{B}}_{d}$ is the destroyed exergy (W).

**Figure 1:**
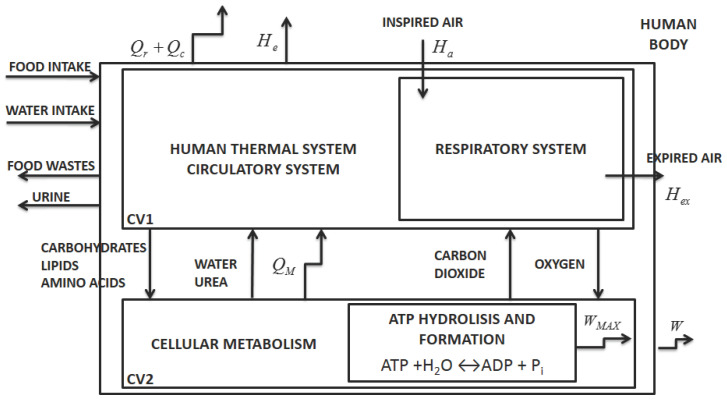
Human body proposed by [5], dividing it into two control volumes: human thermal model (CV1) and cellular metabolism (CV2). Inside the cellular metabolism, there is a reaction of adenosine triphosphate (ATP) formation and hydrolysis. Note that the body is considered as CV1 + CV2, and the surface of the skin is the control volume named the human body.

According to Mady and Oliveira Junior [5], the human body may be simplified as two control volumes, indicated in Figure 1 by CV1 and CV2. The first one represents the thermal system and respiratory system and the second the cellular metabolism. The figure indicates the heat transfer rates associated with convection, ${\dot{Q}}_{c}$, and radiation, ${\dot{Q}}_{r}$, and an enthalpy associated with vaporization (including water leaving the body by diffusion and sweat), ${\dot{H}}_{e}$. There is also an enthalpy variation due to respiration $\Delta {\dot{H}}_{res}$. The sum of these quantities is the energy transfer to the environment (${\dot{E}}_{env}$). Each stream leaving the control volume has its own associated exergy; hence, ${\dot{B}}_{env}={\dot{B}}_{c}+{\dot{B}}_{r}+{\dot{B}}_{e}+\Delta {\dot{B}}_{res}$. The term ${\dot{Q}}_{M}$ is the heat rate released to the body caused by cellular metabolism and will be further analyzed.

Energy and exergy analysis can be applied to the human body (global control volume indicated in Figure 1) resulting in Equations (3) and (4); where ${\left.\frac{dU}{dt}\right|}_{\Delta T}$ and ${\left.\frac{dB}{dt}\right|}_{\Delta T}$ are the energy and exergy variations of the body associated with the variations of internal temperature over time. The terms $\dot{M}$ and ${\dot{B}}_{M}$ are the metabolic internal energy and exergy variations associated with the enthalpy and exergy variations of the reactions of oxidation within the human body. As previously discussed, ${\dot{E}}_{env}$ and ${\dot{B}}_{env}$ are the energy and exergy transfer rates to the environment (associated with heat and mass transfer). The term ${\dot{B}}_{d}$ is the exergy destroyed rate of the body. The unit of the terms of these equation is Watts (W). It is important to state that the energy ($\dot{M}$) and exergy (${\dot{B}}_{M}$) metabolisms are considered as part of $\frac{dU}{dt}$ and $\frac{dB}{dt}$ according to Equations (5) and (6):

(3) $${\left.\frac{dU}{dt}\right|}_{\Delta T}=\dot{M}-\dot{W}-{\dot{E}}_{env},$$

(4) $${\left.\frac{dB}{dt}\right|}_{\Delta T}={\dot{B}}_{M}-\dot{W}-{\dot{B}}_{env}-{\dot{B}}_{d}.$$

The main concern in the present work is the cellular metabolism (ATP hydrolysis and formation). To this aim, Equations (5) and (6) demonstrate the definition of internal energy and exergy variations of the body over time. These are the compositions of the energy and exergy variations of the body associated with the variations of internal temperature over time, previously defined; and enthalpy and exergy variations of the reactions of oxidation of the ingested nutrients. $\dot{M}=-\Delta {\dot{H}}_{nutrients}$ and ${\dot{B}}_{M}=-\Delta {\dot{B}}_{nutrients}.$

(5) $$\frac{dU}{dt}=-\dot{M}+{\left.\frac{dU}{dt}\right|}_{\Delta T}$$

(6) $$\frac{dB}{dt}=-{\dot{B}}_{M}+{\left.\frac{dB}{dt}\right|}_{\Delta T}$$

Therefore, the energy and exergy analyses for Control Volume 2 (CV2) are indicated in Equations (7) and (8). These equations were written for the case where there is no temperature variation over time in CV2. Hence, all variations of temperature over time were considered entirely in CV1. As a consequence, in the body, ${\left.\frac{dU}{dt}\right|}_{\Delta T}=0$, only when the internal temperature is constant.

One important discussion for these equations is the difference of applying the second law of thermodynamics. If there is no performed power (resting condition), the enthalpy variation of the reactions of oxidation equals the heat released to the body from metabolism, but from the exergy analysis perspective, these are two completely different quantities: the order of magnitude is different. The exergy associated with the heat released by metabolism is ${\dot{B}}_{Q_{M}}={\dot{Q}}_{M}\left(1-\frac{T_{0}}{T_{b}}\right)$, where $T_{b}$ is the internal temperature or the muscle (depending on the activity in consideration).

(7) $${\dot{Q}}_{M}={\dot{H}}_{reac}-{\dot{H}}_{prod}-\dot{W},$$

(8) $${\dot{B}}_{dest}^{VC2}={\dot{B}}_{reac}-{\dot{B}}_{prod}-{\dot{B}}_{Q_{M}}-\dot{W},$$

where in Equations (7) and (8), ${\dot{Q}}_{M}$ is the heat released to the body by metabolism, ${\dot{H}}_{reac}-{\dot{H}}_{prod}$ is the enthalpy variation of the reactions of oxidation of the nutrients in the body (glucose, palmitic acid and amino acid) and performed power $\dot{W}$. All the quantities are in Watts (W).

As in the work of Mady and Oliveira Junior [5], there is a need to evaluate which compounds are degraded in human metabolism. Glucose was chosen as a representative of carbohydrates (Equation (9)), palmitic acid as a representative of lipids (Equation (10)) and an amino acid (with an average composition) to represent the proteins (Equation (11)). It is important to highlight that the amino acid does not suffer complete oxidation in the human body; moreover, this pseudo-molecule was proposed based only on compounds that exist in the human body. From these equations, it is interesting to define the RQ (respiratory quotient), which is the ratio of the carbon dioxide production to oxygen consumption (on a molar or volumetric basis). For Equation (9), this quantity is one; for Equation (10), it is 0.7; and for Equation (11), it is 0.83. This index gives a clue as to which nutrient is consumed in each activity or if it is a combination of these molecules.

(9) $$C_{6}H_{12}O_{6}+6O_{2}\to 6CO_{2}+6H_{2}O$$

(10) $$C_{16}H_{32}O_{2}+23O_{2}\to 16{CO}_{2}+16H_{2}O$$

(11) $$C_{4.98}H_{9.8}N_{1.4}O_{2.5}+5.135O_{2}\to 4.28{CO}_{2}+3.5H_{2}O+0.7{CH}_{4}N_{2}O$$

From the stoichiometry of the reactions of oxidation (Equations (9)–(11)), it is possible to evaluate the amount of oxygen consumption (${\dot{m}}_{O_{2}}$) and carbon dioxide production (${\dot{m}}_{{\mathrm{CO}}_{2}}$) obtained from indirect calorimetry. With these data, it is possible to calculate the amount of nutrients that were used to liberate the energy for the activity. Hence, it is possible to obtain the system of equations represented by Equations (12)–(14), as discussed in [5]. It should be noted that ${\dot{m}}_{N}$ is the amount of nitrogen excreted in the urine, and conventionally, each gram of nitrogen excreted in the urine represents the oxidation of 6.45 g of amino acids, as discussed in [37,38]. Furthermore, it was assumed that in one day, there was an excretion of 12 g of nitrogen in the urine [38] due to the oxidation of amino acids.

(12) $$\begin{array}{c} {\dot{m}}_{carb}/1000=-2.14{\dot{m}}_{O_{2}}+2.24{\dot{m}}_{{\mathrm{CO}}_{2}}-3.39{\dot{m}}_{N} \end{array}$$

(13) $$\begin{array}{c} {\dot{m}}_{lip}/1000=1.14{\dot{m}}_{O_{2}}-0.83{\dot{m}}_{{\mathrm{CO}}_{2}}-1.50{\dot{m}}_{N} \end{array}$$

(14) $$\begin{array}{c} {\dot{m}}_{ami}/1000=6.45{\dot{m}}_{N} \end{array}$$

In these Equations, ${\dot{m}}_{carb}$ stands for carbohydrates consumed in metabolism (represented by glucose), ${\dot{m}}_{lip}$ stands for lipids’ consumption in metabolism (represented by palmitic acid) and ${\dot{m}}_{ami}$ amino acids consumed in metabolism. All of these quantities are obtained in indirect calorimetry in g/s and here used in kg/s, justifying the factor of 1000.

From the set of Equations (12)–(14) and coupled with the data of the indirect calorimetry (oxygen consumption and carbon dioxide production), it is possible to evaluate metabolism on an energy and exergy basis as a function of nutrients’ consumption. Equations (15) and (16) indicate the procedure to calculate metabolism on an energy basis and Equations (17) and (18) on an exergy basis as proposed by Mady and Oliveira Junior [5]. It is important to highlight the necessity of having the energy and exergy (or Gibbs free energy) variation of the reaction of oxidation, which were obtained in [5,39,40].

(15) $$-M=\Delta {\dot{H}}_{M}={\dot{m}}_{carb}\Delta h_{carb}+{\dot{m}}_{lip}\Delta h_{lip}+{\dot{m}}_{ami}\Delta h_{ami}$$

(16) $$\dot{M}=11371{\dot{m}}_{O_{2}}+2366{\dot{m}}_{{\mathrm{CO}}_{2}}+6891{\dot{m}}_{N}$$

(17) $$-{\dot{B}}_{M}=\Delta G_{M}={\dot{m}}_{carb}\Delta b_{carb}+{\dot{m}}_{lip}\Delta b_{glic}+{\dot{m}}_{ami}\Delta b_{ami}$$

(18) $${\dot{B}}_{M}=9363{\dot{m}}_{O_{2}}+4444{\dot{m}}_{{\mathrm{CO}}_{2}}+8764{\dot{m}}_{N}$$

Note that Equations (16) and (18) represent metabolism on the basis of energy and exergy; therefore, the unit of the equations is Watts (W). The enthalpy and exergy variation of each nutrient (kJ/kg or J/g) multiplied by the mass consumption of these nutrients (kg/s or g/s) is calculated in Equations (12) to (14).

As discussed in [5], according to Nelson and Cox [41], the degradation of carbohydrates, lipids and proteins in human cells occurs gradually with the contributions of several enzymes to reduce the activation energy of the reactions. Thus, the energy is gradually captured with a certain efficiency, adding an inorganic phosphate group (P${}_{i}$) to adenosine diphosphate (ADP) to form adenosine triphosphate (ATP) according to Equation (19). The reverse equation is the ATP hydrolysis, and it is responsible for any energy conversion process in human cells. In other words, to perform any kind of work, the human body obtains energy from the reverse reaction known as ATP hydrolysis ($\Delta g_{0}^{{}^{\prime }}$ = −30.5 kJ/mol in the standard biochemical reference).

It is important to comment that the actual biological condition is not the same as the actual condition of the human body. The authors in [4,41,42,43] proposed modifications of the reference that account for the effects of reactant and product concentrations, acid and base dissociation, free magnesium ion interaction, ionic interactions, effects of electrical potential, and so on. Based on these authors’ results, the actual free energy change of ATP hydrolysis ($\Delta g_{ATP}$) in the reverse Equation (19) is −56 kJ/mol. Note that although the nutrients are obtained and delivered to the reference environment, the molecule of ATP only exists inside the body, justifying these modifications for the real conditions in which the ATP formation and hydrolysis occur.

(19) $$ADP+P_{i}⟷ATP+H_{2}O$$

Several authors [5,11,12,30,34,44] used Equation (20) to evaluate the metabolic efficiency of each nutrient and type of oxidation:

(20) $$\eta_{M}=\frac{\Delta G_{ATP}}{\Delta G_{OXI}}=\frac{\Delta G_{ATP}}{B_{M}}.$$

From [5,40,41], after the complete aerobic oxidation of the nutrient in the cells, a certain quantity of ATP is formed. One mole of glucose is responsible for the formation of 32 moles of ATP; one mole of palmitic acid, 106 moles of ATP; and 1 mole of amino acid, 8 moles of ATP (this last value was obtained in [11,12] and used in [5]). Bearing this in mind, Equation (21) can be expressed as a function of ATP production/hydrolysis or for each nutrient, as a function of the results of the calorimetric system (Equations (12)–(18)).

**Figure 2:**
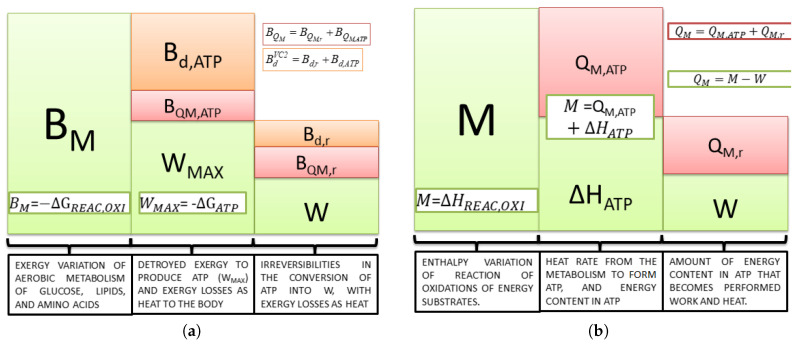
(**a**) Exergy conversion process in the cellular metabolism. From nutrient oxidation, ATP formation and the use of ATP. (**b**) Energy analysis of the conversion of the chemical energy of substrates into work and heat. Obtained and modified from [36].

Equation (21) is important because it states the maximum available work of the body; independent of the nutrient ingested, ATP is the only compound that the body uses to obtain energy for any kind of activity. Therefore, to produce ATP, there must be some irreversibilities, as seen in Figure 2.

(21) $${\dot{W}}_{max}={\dot{n}}_{ATP}\left|\Delta g_{ATP}\right|=\sum_{i=1}^{3}\eta_{M,i}\left|\Delta {\dot{G}}_{i,OXI}\right|$$

Furthermore, it is possible to evaluate the moles of ATP hydrolyzed as a function of the carbohydrate, lipid and amino acid consumption rate, as in Equation (22).

(22) $$n_{ATP}=32\cdot \frac{m_{carb}}{M_{glic}}+106\cdot \frac{m_{lip}}{M_{lip}}+8\cdot \frac{m_{amin}}{M_{amin}}$$

It is important to highlight that only from the second law perspective is it possible to make a statement regarding the maximum performed work. The first law guarantees that the energy is conserved and transferred as heat, work and enthalpy. Moreover, with these results, the exergy analysis may be used to support biomechanics, as stated in [29], with an upper limit to the existing equations and methods to evaluate the performed work.

In Figure 2b, the energy released from the oxidation of nutrients (i.e., carbohydrates, lipids and proteins) (*M*) can be only transformed into heat ($Q_{M}$) and work (*W*). In addition, Figure 2b demonstrates that even evaluating the energy variation of Reaction (19), the information obtained is the amount of energy released as heat (dissipated to the rest of the body). Moreover, it is impossible to state the value of the maximum available work.

Figure 2a demonstrates the exergy analysis for the same steps of metabolism, where $B_{M}$ is the exergy variation of the reactions of oxidation of nutrients (maximum work that could extract the body of nutrients consumed). Since this kind of study takes the quality of the energy conversion in each process into consideration, it is possible to calculate the maximum available work from the Gibbs free energy of ATP hydrolysis ($W_{MAX}=-\Delta G_{ATP}$) and the destroyed exergy to produce this molecule from $ADP+P_{i}$. Equation (23) describes the amount of exergy destroyed in metabolism to obtain a certain amount of ATP degrading the macronutrients. The remainder from the first law point of view is released as heat, represented on an exergy basis as ${\dot{B}}_{Q_{M,ATP}}$:

(23) $${\dot{B}}_{d,ATP}={\dot{B}}_{M}-{\dot{B}}_{Q_{M,ATP}}-{\dot{W}}_{MAX}.$$

The difference between maximum available and performed work (or power) quantifies the inefficiencies among the process of ATP utilization demonstrated in the third column of the exergy conversion process. The destroyed exergy in the processes are $B_{d,ATP}$ and $B_{d,r}$, and the exergy lost as heat is $B_{Q_{M,ATP}}$ and $B_{Q_{M,r}}$. The first term is calculated as $B_{Q_{M,ATP}}=Q_{M,ATP}\left(1-T_{0}/T_{b}\right)$ and the second $B_{Q_{M,r}}=Q_{M,r}\left(1-T_{0}/T_{b}\right)$. It is important to state that if a computational model is used such as in [45], $T_{b}$ may be evaluated as an average temperature, whereas in experimental results, the tympanic or rectal temperature are commonly used as representative of the internal temperature of the body.

From these two figures, it is possible to conclude that if there is no performed power, all the energy released in metabolism becomes heat, as in Equation (7) (Figure 2b); however, from Equation (8), part of the exergy content in the nutrients is destroyed, and the remainder is released as exergy associated with heat (Figure 2a).

It is interesting to discuss at this point the possibility of the definition of three types of efficiency. The first one is the ratio of $W_{MAX}$ to $B_{M}$, which is defined as the metabolic efficiency (Equation (20)). The second is the ratio of *W* to $W_{MAX}$ and the third *W* to $B_{M}$. The former is more often used in the literature to evaluate the efficiency of real work, and the latter has a similar trend and is simpler to evaluate. Therefore, the second step (second to third column) is evaluated from the exergy optics using Equation (24). Note that there is an amount of exergy not converted into power, which is lost as heat. If the conversion efficiency were 100%, there would neither be destroyed exergy, nor heat released in this step.

(24) $${\dot{B}}_{d,r}={\dot{W}}_{MAX}-\dot{W}-{\dot{B}}_{Q_{M,r}}$$

This discussion is done in [44] from different perspectives, where the efficiency values of the isolated muscles of different animals were obtained. Based on the results of [46], there is even an efficiency for human muscle cells for some restricted conditions. For an isolated muscle, the efficiency ranges from 0.14 to 0.28 (environment of 12 to 20 ${}^{\circ }$C). Other authors [47] found the overall efficiency for skeletal muscle ranging from 0.17 to 0.42.

In the case of anaerobic metabolism, the reaction occurs in the body during strenuous activities. In this condition, the only chemical compound used is glucose, with the formation of two molecules of lactic acid ($C_{3}H_{5}O_{3}$) and two molecules of ATP. This reaction is indicated by Equation (25). It is important to highlight that this is a way found by the body to use energy quickly in the muscle, although with low efficiency, since the lactic acid has a high exergy content. The maximum available work from this reaction (therefore, the Gibbs free energy variation or exergy variation) is −226.4 kJ/mol.

(25) $$C_{6}H_{12}O_{6}\to 2C_{3}H_{6}O_{3}$$

Taking only glucose metabolism into account, Table 1 indicates the difference between the exergy variation of these reactions of oxidation. It is expected that anaerobic reactions would be faster, although they release less exergy per mole of glucose. Hence, the metabolic efficiency would be lower, but this is the only way for the body to obtain energy quickly without the presence of oxygen. The total enthalpy variation of the complete oxidation of glucose is −2872 kJ/mol, as demonstrated in [5,40,41]. Consequently, the fourth column of Table 1 indicates the metabolic efficiency of these two types of oxidation. It is evident that although the anaerobic respiration is faster, its efficiency is an order of magnitude lower than the aerobic one.

## 3. Experimental Procedure

From the previous studies, it was stated that depending on the equation used to evaluate the performed work on a treadmill, the exergy analysis would result in different efficiencies and destroyed exergy [36]. Nevertheless, from the analyses proposed, the exergy-based indexes were demonstrated to be a potential tool concerning to discriminate the subjects as a function of their training level [29]. Better-trained subjects seem to use the exergy content in ATP more efficiently than the ones at a lower training level. In the present article, the performed power is a known variable aiming at the possibility to assess the exergy efficiency of different exercises of the human body. There was no intent to compare with medical indexes (maximum oxygen consumption and lactate threshold) as performed in [29], justifying the usage of only two subjects. For the sake of simplicity, there was no necessity to perform a statistical analysis with several subjects and measures. The final purpose of the article is to choose between weight lifting and stationary bicycle in pursuance of replicating the experiments of [29].

A distinguishing feature of this article is that based on these previous studies, two subjects were analyzed performing two activities in which the performed power was already known. The room temperature was 22.4 ${}^{\circ }$C and the relative humidity 73% (these were considered the reference temperature $T_{0}$ and $\phi_{0}$ for the application of the exergy analysis). In both cases, the performed work (or power) was calculated according to the definition of performed work, which is: “work is done by a system on its surroundings if the sole effect on environment external to the system could be characterized by a rising of a weight”. These experiments were:

1 Weight lifting (biceps curl): a continuous series of lifting (76 repetitions) was performed and with one arm (the forearm is considered to elevate the mass to 0.26 m) and a mass of 4 kg until the exhaustion of the subject. This was an experimental protocol aiming at the proposition of the most suitable exercise to apply the exergy analysis.
2 Stationary bicycle, where there was an incremental cadence of the bicycle (Wattbike, Model Pro/Trainer) every 4 min. When the subject was exhausted, the level of activity decreased, and the exercise continued only for recovery purposes, which was around 17 min of activity. This protocol was based on the one proposed in [29]. The performed power was obtained directly from the bicycle, taking into account the thermodynamic definition.

The experimental procedure was approved by the Ethics Committee for Analysis of Research Projects (CAPPesq-Registered Number 16507) of the Faculty of Medicine of the University of São Paulo.

A calorimetric system (Medgraphics, Model CPX/Ultima) was applied to evaluate the O${}_{2}$ consumption and the CO${}_{2}$ production in order to calculate metabolism. The data were acquired in each breath, and average values for each minute were calculated. According to the manufacturer, the accuracy of the flow sensor is ±3% or 50 mL (whichever is greater); the accuracy of the O${}_{2}$ galvanic sensor is ±1%; and the accuracy of the CO${}_{2}$ non-dispersive infra-red sensor is ±0.1%. Before each test, the equipment was calibrated with a 3-L syringe for the flow rate and with cylinders containing known fractions of O${}_{2}$ (12%) and CO${}_{2}$ (5%).

**Figure 3:**
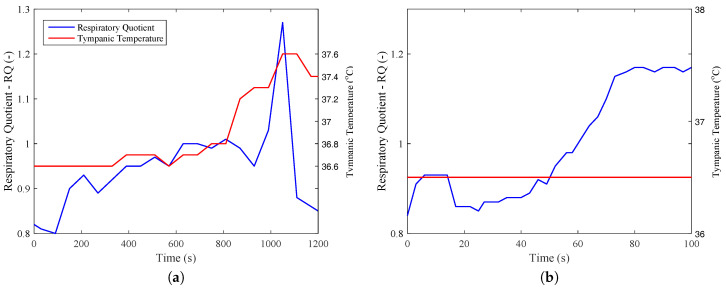
(**a**) Respiratory quotient and tympanic temperature of a subject under a bicycle test, (**b**) respiratory quotient of a subject performing a continuous biceps series with 4 kg (the internal temperature was considered constant, equal to 36.5 ${}^{\circ }$C.

From these data, it was possible to calculate metabolism on an energy and exergy basis from Equations (16) and (18). From Figure 3, it was also possible to obtain the respiratory quotient for the two exercises, where it is clear the it achieves values larger than unity after 18 min for Figure 3a (beginning of recovery) and after one minute for Figure 3b. This figure demonstrates that the exercises have different natures, the first being mostly aerobic and the former anaerobic.

In order to use a representative temperature of the body, the tympanic temperature was measured in the exercise with an ear thermometer (G-Tech). Moreover, the skin temperature was measured (FLIR Camera, model E60), but these values were not used in the present analysis.

**Figure 4:**
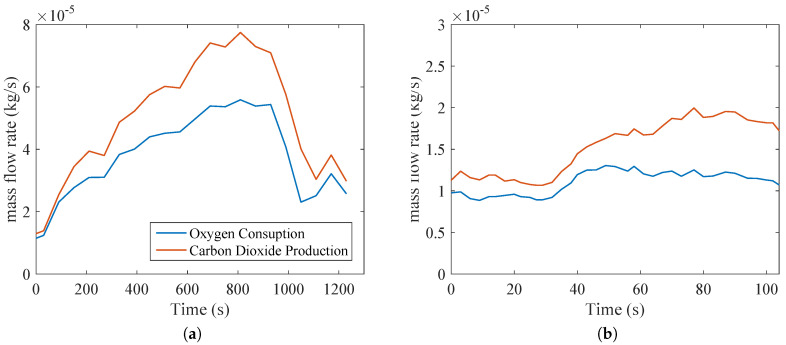
Physiological data collected during tests: oxygen consumption and carbon dioxide production of a subject under: (**a**) bicycle test; (**b**) biceps series.

Figure 4 indicates the oxygen consumption and carbon dioxide production in kg/s to better elucidate the calorimetric data. It must be pointed out that the respiratory quotient is the ratio of carbon dioxide production to oxygen consumption using values of moles/s (or volume). It is important to compare the carbon dioxide production in Figure 4a, which is about four times higher than in Figure 4b, demonstrating that this type of exercise uses a larger musculoskeletal group (legs and thighs muscles, whereas biceps tests uses smaller muscles of the arm).

## 4. Results and Discussion

**Figure 5:**
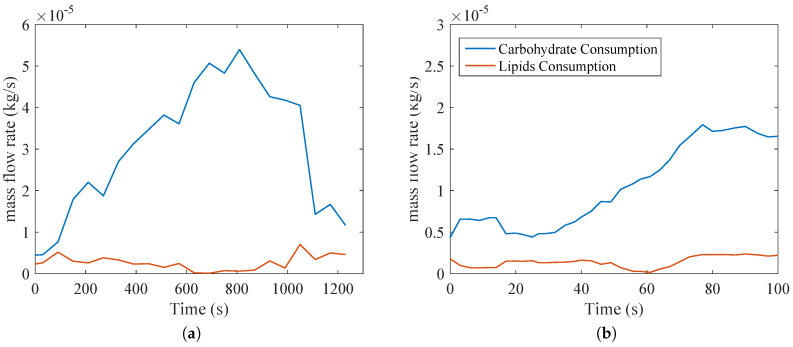
Carbohydrate and lipid consumption during: (**a**) bicycle test; and (**b**) biceps curl. Both cases were obtained using Equations (12) and (13) for the results of Figure 4.

Figure 5 demonstrates the relevance of glucose in the exercise, since according to Equations (12) and (13), most of the nutrient consumption is of this one. This may raise a future question with respect to the nature of anaerobic metabolism and how it affects both types of activities. When compared with Figure 4, where RQ is close to unity (indicating that the majority of oxidation is of glucose), Figure 5 confirms these trends.

**Figure 6:**
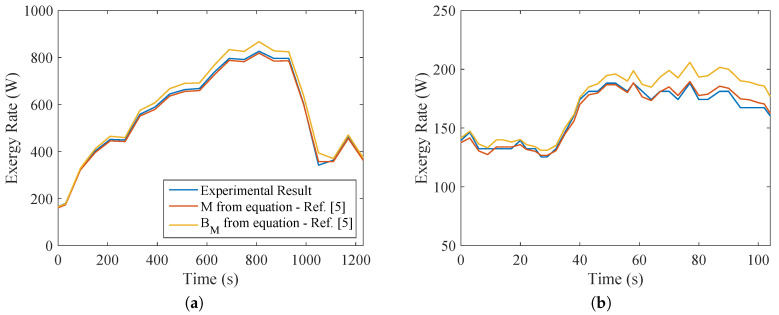
Metabolism obtained from the equipment, metabolism calculated by Equation (16) and exergy metabolism calculated from Equation (18). (**a**) Bicycle test; (**b**) weight lifting.

From the results of the indirect calorimetry, it was possible to evaluate, for both types of exercise, the metabolic energy and exergy and compare with the values given by the equipment. This is indicated in Figure 6. The same conclusions of [5,13,29,37] were reached, that there is no significant difference in the values of *M* and $B_{M}$. Moreover, a more intensive exercise would result in higher values of metabolism. This becomes clear when comparing both types of exercise. The difference in metabolism during the test was one order of magnitude, demonstrating that this type of exercise uses a larger musculoskeletal group (legs and thigh muscles, whereas biceps tests use smaller muscles of the arm).

**Figure 7:**
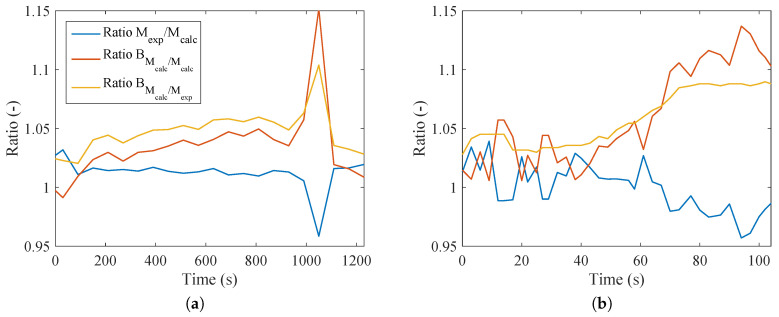
Ratio of $M_{exp}$ to $M_{calc}$, $B_{M_{calc}}$ to $M_{calc}$ and $B_{M_{calc}}$ to $M_{exp}$, for: (**a**) bicycle test; (**b**) weight lifting.

Figure 7a indicates that the ratio of the different methods to calculate metabolism was no larger than 5%. At only one point was there a difference of about 15%. This point is where a deceleration occurs and the subject continues to exercise, but with lower intensity (recovery period). In the case of Figure 7b, the trend was not as smooth, and comparing with Figure 3b, it is possible to conclude that higher values of the respiratory quotient resulted in higher ratios of metabolism on an energy and exergy basis. Therefore, for these cases, the assumption of $M\approx B_{M}$ is not valid. This result contradicts most of the literature in this area; nevertheless, this kind of test has not yet been performed, and further investigations should be done in order to better evaluate this behavior (since a divergence occurred at only one point).

**Figure 8:**
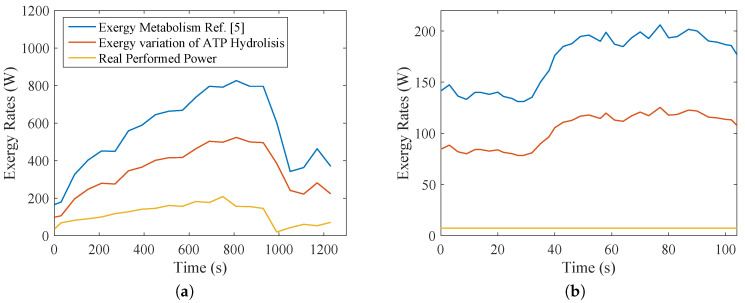
Exergy terms indicating the metabolic efficiency, from the transformations of $B_{M}$ (Equation (18)) into ATP (ratio of $W_{MAX}$ to $B_{M}$) and ATP into Wreal (ratio of $W_{real}$ to $W_{MAX}$) for the two studied cases: (**a**) bicycle test; (**b**) weight lifting.

Figure 8a,b demonstrates a very interesting result regarding the metabolic efficiency and the necessity of the body to modify the carbohydrates, lipids and amino acids in only one type of substance (ATP), with its hydrolysis being the maximum available work to the human body ($W_{MAX}$). Eventually, it was possible to evaluate the exergy content in ATP to performed power (*W*).

From the data collected, it was possible to evaluate the metabolic exergy, the maximum available work in ATP molecules and the real performed power. One conclusion with the comparison of Figure 8a,b is that the efficiency of aerobic exercise is higher than weight lifting. Nevertheless, this should be read carefully, because a larger amount of muscle area was used in Figure 8a, increasing the metabolic exergy and maximum available work in order to perform the required power. On the other hand, the increase in the total metabolism of the body for Figure 8b is very low due to the size of the biceps and the anaerobic nature of the activity. A lower performed power by the muscle in comparison to the overall metabolism of the body is expected, therefore justifying its lower exergy efficiency.

**Figure 9:**
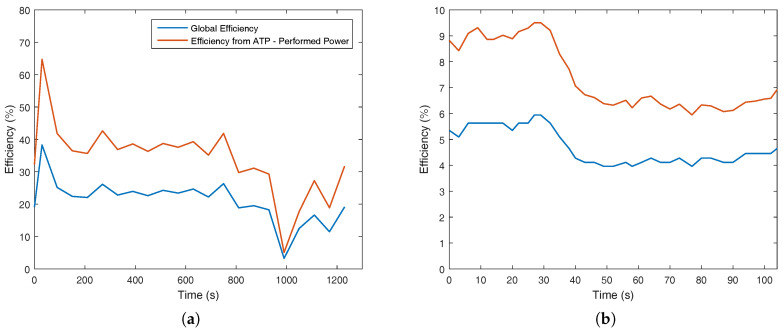
Global efficiency (ratio of *W* to $B_{M}$) and the efficiency of the conversion of ATP into $W_{real}$ (ratio of $W_{real}$ to $W_{MAX}$), for two cases (ratio of $W_{MAX}$ to *W*): (**a**) bicycle test; (**b**) weight lifting.

One other perspective that must be taken into account is how the body degrades the nutrient molecules, where a higher amount of aerobic metabolism leads to higher efficiency. A careful analysis of Figure 3b may indicate that when the RQ (respiratory quotient) has its magnitude larger than unity, there is the presence of anaerobic metabolism, which justifies lower efficiencies for Figure 8b than Figure 8a. These results may be evaluated using Figure 9, where the efficiency of Figure 9a is around three-times higher than the efficiency of the biceps test (Figure 9b).

**Figure 10:**
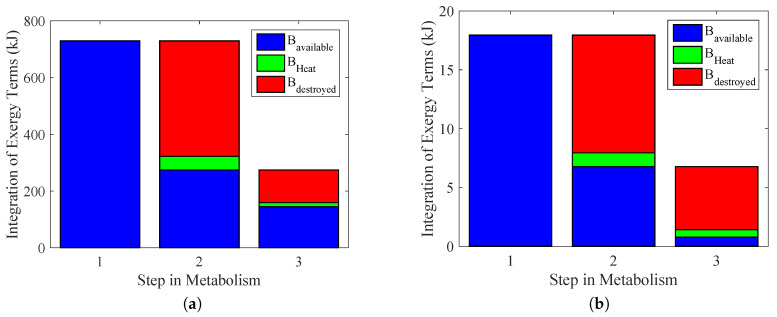
Exergy conversion process in cellular metabolism. From nutrient oxidation, ATP formation and the use of ATP. Based on the figures proposed in [36]. (**a**) Bicycle test; (**b**) weight lifting.

Figure 10 summarizes the results of previous figures and indicates the efficiency of the energy conversion, as well as the exergy transfer associated with heat and the irreversibilities in each path of metabolism (therefore the degradation of nutrients) to performed work for some activity. The importance of the results of Figure 10a,b is that instead of evaluating different performed power as done in [29,36], the main objective was to choose the variables to measure. In this figure, it was possible to evaluate each step of metabolism, but with a better precision.

1. The first column (1) indicates the maximum work that the body could extract from nutrients; therefore, the metabolic exergy (in blue). In order to obtain these values, the exergy metabolism was integrated over the period of time of the test. The value obtained in Figure 9a was 730 kJ. For Figure 9b, it was 18 kJ.
2. Because the body only consumes ATP as a nutrient to perform any physical activity, there must be some irreversibilities in this step, as indicated in Column (2), using an integration of Equation (21). This result is in accordance with [5]. Nevertheless, there is a destroyed exergy in the process, in red ($B_{d_{ATP}}$), and an exergy loss associated with heat, in green ($B_{Q_{ATP}}$).
3. In the last step, Column (3), there is the energy conversion process from the ATP to actual performed power in blue, with its exergy lost as heat ($B_{Q_{r}}$) and destroyed ($B_{d_{r}}$). These were obtained by an integration of Equations (23) and (24).

**Figure 11:**
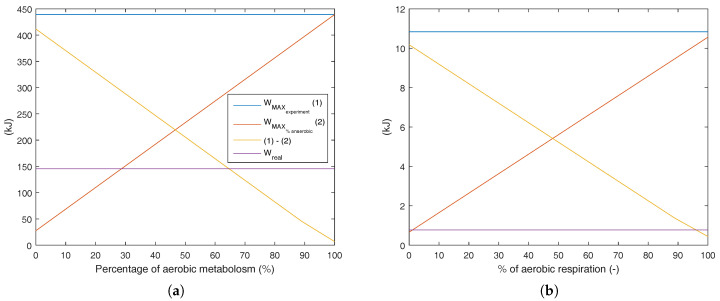
Exergy analysis of the conversion of the chemical exergy of substrates into work and heat. Several metabolic efficiencies were evaluated, from 100 % of aerobic to 100 % of anaerobic oxidation. (**a**) Bicycle test; (**b**) weight lifting.

When the respiratory quotient is higher than unity, it is supposed to have a higher percentage of anaerobic metabolism. Since it is not possible to properly define the amount of anaerobic metabolism only for calorimetry results, Figure 11 indicates all possible combinations of aerobic and anaerobic metabolism, and the results were integrated over time. To this aim, using each percentage of aerobic exercise as an input for the whole test, it is possible to evaluate which amount of anaerobic respiration is not possible, and therefore violates the second law of thermodynamics.

In Figure 11b, it is possible to note that weight lifting had a higher influence on the anaerobic metabolism and that there was a violation of the second law of thermodynamics only if 100% of the total energy were obtained through Reaction (25). For the case of the aerobic exercise, where a larger muscle group was used, there was entropy generation lower than zero if 30% of metabolism was from Reaction (25). It must be stated that the real metabolism was measured, although there is no available method in the literature that estimates the amount of aerobic and anaerobic reactions in a physical activity (only with calorimetric data). The idea of these figures is to evaluate an upper limit for these kinds of activities.

**Figure 12:**
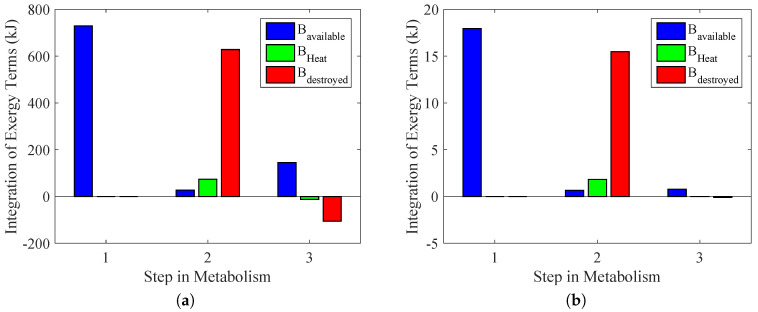
Extreme case where all of metabolism is considered as anaerobic. The first group of columns is the exergy conversion process in the cellular metabolism. From nutrient oxidation, ATP formation and the use of ATP. Based on the figures proposed in [36]. (**a**) Bicycle test; (**b**) weight lifting.

Figure 12 demonstrates these results, but for each step in the reaction chain. In an extreme case where there is no aerobic metabolism, it is possible to conclude that both types of exercise violate the second law of thermodynamics. The performed power is the same as Figure 10, although the destroyed exergy was lower than zero. One important issue is that this represents a theoretical result in which there is no presence of aerobic reaction, and therefore, it is only an illustration of why the second law of thermodynamics may contribute to properly evaluating the amount of aerobic and anaerobic reactions without the necessity of measuring the lactate in the blood, for instance.

## 5. Concluding Remarks

In this work, a combination and extension of different previous works of the group was performed to evaluate the quality of the energy conversion process in the human body. Two scenarios were evaluated with two subjects (there was no intention to produce a statistical analysis, but to apply and validate the measurement method for future analyses). Two types of exercises were evaluated, the first being an increment in the cadence of the bicycle (Wattbike, Model Pro/Trainer), and in the second exercise, a continuous weight lifting series was performed (biceps curl with one arm, 4 kg). A distinguishing feature of this article is the previous knowledge of the performed power in both scenarios. From the range analyzed, it was possible to conclude that:

- The bicycle test is more efficient than weight lifting, from the second law perspective. Nevertheless, this result must be understood with some care, since larger muscular groups are used, although the nature of metabolism is different.
- The metabolic path, from nutrients’ consumption (obtained by indirect calorimetry) to performed power, was first analyzed from the exergy analysis point of view.
- The exergy efficiency achieved values around 40% if the exergy input considered was the ATP and values around 30% if the complete cycle was evaluated ($B_{M}$ as exergy input).
- The exergy efficiency was no larger than 10% for the weight lifting.
- If all of the metabolism was anaerobic, both cases would violate the second law of thermodynamics. This last result demonstrates the characteristic of this kind of nutrient degradation: fast energy conversion, although with low efficiency (use less exergy from the nutrient).
- The most important conclusion is that for future tests involving the application of the first and second laws of thermodynamics, the stationary bicycle test is adequate. It is more precise in the definition of performed power, even when compared with the treadmill, as indicated [29,36]. In future experiments, the group will focus on the referred exercise.

## Figures and Tables

**Table 1:** Aerobic and anaerobic reactions of the oxidation of glucose, Gibbs free energy variation and the amount of ATP produced [5,40,41].

Type of Reaction	Moles of ATP	$\Delta g_{0}^{{}^{\prime }}$ (kJ/mol)	$\eta_{M}$ (%)
Aerobic Equation (9)	32	−2872	62.4
Anaerobic Equation (25)	2	−226.4	3.90
